# Examination of the Myokine Response in Pregnant and Non-pregnant Women Following an Acute Bout of Moderate-Intensity Walking

**DOI:** 10.3389/fphys.2019.01188

**Published:** 2019-10-10

**Authors:** Kelly Ann Hutchinson, Shuhiba Mohammad, Léa Garneau, Kurt McInnis, Céline Aguer, Kristi B. Adamo

**Affiliations:** ^1^Faculty of Health Sciences, School of Human Kinetics, University of Ottawa, Ottawa, ON, Canada; ^2^Recherche, Institut du Savoir Montfort, Ottawa, ON, Canada; ^3^Department of Biochemistry, Microbiology and Immunology, Faculty of Medicine, University of Ottawa, Ottawa, ON, Canada

**Keywords:** pregnancy, myokines, physical activity, exercise, gestational weight gain

## Abstract

**Background:**

It is recommended that women accumulate 150-min of weekly moderate-intensity physical activity (MPA) when pregnant. Engaging in regular physical activity (PA) confers many health benefits to both the mother and the fetus. However, the molecular mechanisms by which these health benefits are bestowed are not well understood. One potential factor that may be contributing to the observed benefits is myokines, which are small peptides secreted by skeletal muscles. In the non-pregnant population, myokines are believed to be involved in the molecular mechanisms resulting from PA. The objective of this study was to characterize and compare the myokine profile of pregnant and non-pregnant women, after an acute bout of MPA.

**Methods:**

Pregnant (*n* = 13) and non-pregnant (*n* = 17) women were recruited from the Ottawa region to undergo a treadmill walking session at moderate-intensity (40–60% heart rate reserve). Pre- and post-exercise serum samples were taken, and a set of 15 myokines were analyzed although only 10 were detected. IL-6 was analyzed using a high-sensitivity assay, while FGF21, EPO, BDNF, Fractalkine, IL-15, SPARC, FABP-3, FSTL-1, and oncostatin were analyzed using various multiplex assays.

**Results:**

The pregnant and non-pregnant groups did not differ in terms of age, height, non/pre-pregnancy weight, BMI, and resting heart rate. Baseline levels of EPO and oncostatin were higher in the pregnant group while FGF21 was higher in the non-pregnant group. Circulating levels of three myokines, FGF21, EPO, and IL-15 significantly increased in response to the acute exercise in the pregnant group. Non-pregnant women exhibited an increase in three myokines, FABP-3, FSTL-1, and oncostatin, while one myokine, EPO, decreased post-exercise. SPARC, fractalkine and BDNF were shown to increase post-exercise regardless of pregnancy status while the response for BDNF was more pronounced in the non-pregnant group.

**Conclusion:**

This is the first study examining myokine response following an acute bout of PA in pregnancy. Moderate intensity PA, which is recommended during pregnancy, elicited an increase in four myokines post-compared to pre-exercise in the pregnant group. Further research is warranted to understand the role of myokines in pregnancy.

## Introduction

Contrary to outdated beliefs, evidence shows that engaging in regular physical activity (PA) during pregnancy is associated with a plethora of health benefits for both the mother and the fetus (Nascimento et al., 2012; Mudd et al., 2013). The 2019 Canadian guidelines for PA throughout pregnancy recommend that pregnant women without contraindications engage in at least 150-min moderate-intensity PA per week (Mottola et al., 2018). A combination of resistance and aerobic exercises has been deemed safe and is highly recommended to achieve greater benefits (Mottola et al., 2018). Systematic review evidence illustrates that pregnant women who are physically active compared to those that are not experience less musculoskeletal pain, gestational weight gain (GWG), postpartum weight retention, urinary incontinence as well as gestational diabetes and insulin resistance (Nascimento et al., 2012; Davenport et al., 2018a, b; Ruchat et al., 2018). Exercise interventions during pregnancy have been shown to decrease the odds of developing gestational hypertension and pre-eclampsia (Davenport et al., 2018c). Additionally, habitual PA while pregnant can decrease depressive symptoms during pregnancy and the postpartum period (Robledo-Colonia et al., 2012; Vargas-Terrones et al., 2018) and improve quality of life (Victoria et al., 2010). Alternatively, PA engagement during pregnancy may have positive downstream effects for the health of the infant (Ferraro et al., 2012). These findings are in line with the developmental origins of health and disease (DOHaD) hypothesis, suggesting that the intrauterine environment plays a critical role in determining health outcomes later in life (Wadhwa et al., 2009). Being physically active during pregnancy could contribute to optimizing the development of the fetus’ immune and central nervous systems, thereby decreasing the risk of developing neurodevelopmental and psychiatric disorders (Marques et al., 2015). It is possible that maternal-fetal health benefits linked to PA may be accorded through changes in development and function of the placenta. Regular participation in PA throughout pregnancy is associated with improved placental function by way of optimizing nutrient transport to the fetus due to an increase in intervillous space blood volume (Jackson et al., 1995; Clapp, 2003).

Although it is well established that habitual PA is beneficial during pregnancy, the molecular mechanisms by which PA acts on different organs and body systems in pregnant women remain to be fully understood. It has been suggested that myokines, peptides that are synthesized by skeletal muscles and most often released in the body as a result of contraction (Pedersen et al., 2007), are responsible, in part, for the crosstalk between muscles and various organs and tissues in the body (Pedersen, 2013). The investigation of myokine response as a result of PA has been mainly conducted using male subjects. To date, the circulating myokine profile resulting from PA has yet to be examined in the context of human pregnancy. Dubé et al. (2017) postulate that myokines released during PA may play a role in the optimization of fetal and placental growth outcomes. Hundreds of these peptides have been identified, including, IL-6, IL-15, and fibroblast growth factor 21 (FGF21) (Leal et al., 2018). Myokines are involved in paracrine and endocrine signaling pathways (Pedersen, 2013), while some myokines, such as myostatin and leukemia inhibitory factor (LIF) exert their actions in an autocrine fashion upon the same muscle that synthesizes them (Pedersen, 2013; Carson, 2017). Most myokines are not exclusively produced and secreted by muscle fibers but can also be expressed or derived from other tissues or organs in the human body, such as bone, liver, adipose tissue and macrophages (Ishimi et al., 1990; García et al., 1999; Kakoti and Goswami, 2013; Salminen et al., 2017). Collectively, the myokine secretome has various functions attributable to the unique activity of each myokine. For instance, IL-6, the most well-characterized myokine, has been recognized as a key mediator of glucose metabolism as it increases insulin sensitivity (Steensberg et al., 2000). Although the function of each myokine is specific, it is suggested that there is one commonality in their roles, that of being mediators to the benefits and protective effects observed as a result of engaging in PA (Benatti and Pedersen, 2015; Whitham and Febbraio, 2016). However, the characterization of the myokine profile and thus its potential effects, in pregnant women, has yet to be investigated. This study aimed to compare the myokine response to moderate-intensity exercise between non-pregnant and pregnant women.

## Materials and Methods

### Ethics Approval and Informed Consent

This study was approved by the Research Ethics Board at the University of Ottawa (file number: H-06-18-634), and all aspects conform to the Declaration of Helsinki. Written informed consent was obtained from each participant willing and eligible to participate.

### Participants

Pregnant and non-pregnant women were recruited from the Ottawa region (ON, Canada) via recruitment flyers posted at the University of Ottawa and on social media platforms. Eligibility was confirmed via telephone by the researchers. Inclusion criteria were as follows: between 18 and 40 years of age, having a self-reported non/pre-pregnancy body mass index (BMI) classified as normal or overweight (18.5–29.9 kg/m^2^) with no contraindication to exercise. Those with hypertension, diabetes, or untreated thyroid disease were excluded. Pregnant women in their second trimester (13–28 weeks gestation) were included. Participants were excluded if they were not able to complete the exercise session or if a blood sample was not obtained either pre- or post-exercise. Anthropometric measurements such as height and body weight were recorded at the time of the visit using a Tanita HR-200 wall-mounted stadiometer (Lachine, QC) and a Tanita BWB-800 scale, respectively. In the pregnant group, GWG was calculated by subtracting the weight measured at the study visit by the self-reported pre-pregnancy weight. Based on the Institute of Medicine (IOM) recommendations for GWG, women should gain a maximum of 9.7 kg in their first trimester, regardless of BMI (American College of Obstetricians and Gynecologists, 2013). Thereafter, a maximum of 2.2 and 1.5 kg of weight gain per week is recommended for women with a BMI classified as normal and overweight, respectively (American College of Obstetricians and Gynecologists, 2013). The GWG at the time of the visit in addition to gestational age of participants were used to calculate the percentage of upper-limit of weight gained in accordance to the IOM guidelines.

### Exercise Protocol

Participants were asked to fast for 8 h and refrain from any PA for 12 h before the study visit. Upon arrival, participants were provided with, and asked to consume a standardized snack of approximately 340 kcal. The snack consisted of a fruit juice (orange or cranberry), a granola bar and a small fruit (apple or pear). However, we could not force a participant to eat if they chose not to. Following the snack, a 10-min seated resting phase began during which heart rate (HR) was monitored continuously and was recorded at 1-min intervals using a Polar V800 (Lachine, QC) heart rate monitor. Resting HR (RHR) was determined from the average of the last 5-min of measurements. The acute bout of exercise was conducted using a Woodway Pro XL 27 treadmill (Woodway USA, Waukesha, WI, United States) following the resting phase. Initially, participants underwent the acclimation phase starting with a warm-up for 3-min at 2.0 mph followed by an increase in the speed of 0.2 mph every minute, until the calculated moderate intensity, or 40–60% heart rate reserve (HRR), was achieved. The incline was set at 6% throughout both the acclimation and the acute bout of exercise. HRR was calculated using the Karvonen formula (Eqs. 1 and 2) (She et al., 2014).

(1) %HRR=[[HRmax-RHR] % intensity*]+RHR

(2) $${\text{HR}}_{\text{max}}=220-a\,g\,e$$

Once the target HR intensity was met, the speed was kept constant for 30-min. HR was monitored throughout to ensure the target HR intensity zone was maintained. If HR was either below or above the desired range, the speed was adjusted by 0.2 mph accordingly. The rate of perceived exertion (RPE) was measured every 1-min during the acclimation phase and every 5-min during the acute exercise bout using the Borg Scale (Borg, 1982).

### Blood Collection and Processing

A blood sample was taken pre- and post-exercise from the medial cubital vein and collected in serum blood collection tubes (#367820; BD Biosciences, Franklin Lakes, NJ) immediately before and after the completion of the exercise protocol. Serum was left to clot at room temperature for 30-min after which it was centrifuged for 15-min at 4°C at a speed of 1000 × *g* using an Eppendorf 5702R centrifuge (Thermo Fisher Scientific Inc., Mississauga, ON, Canada). Serum samples were stored at −80°C until further analysis.

### Human Myokine Assays

Serum samples from the pregnant and non-pregnant participants were assayed in duplicate for 15 myokines: apelin, brain-derived neurotrophic factor (BDNF), erythropoietin (EPO), fatty acid binding protein 3 (FABP-3), follistatin-like 1 (FSTL-1), fibroblast growth factor 21 (FGF21), fractalkine, interleukin-6 (IL-6), interleukin-15 (IL-15), irisin, LIF, myostatin, oncostatin M, osteocrin and SPARC. Of the 15 myokines analyzed, 10 were detected in our samples. Serum samples were shipped overnight, on dry ice to Eve Technologies (Calgary, AB) for analysis using the MILLIPLEX MAP Human Myokine Magnetic Bead Panel (HMYOMAG-56K, Millipore Sigma, Oakville, ON, Canada). Only four of the fifteen myokines were within the range of detection of the Milliplex assay: oncostatin, FABP-3, FSTL-1, and SPARC. Apelin, BDNF, EPO, FGF21, fractalkine, IL-6, IL-15, irisin, IF, myostatin and osteocrin were undetected in our samples. A custom high sensitivity IL-6 assay (Millipore Sigma) was conducted by Eve Technologies. Additionally, a U-PLEX Assay from Meso Scale Discoveries (MSD, Rockville, MD, United States) was used to analyze five myokines: BDNF, EPO, FGF21, Fractalkine, IL-15 while SPARC was re-analyzed using an R-PLEX assay (MSD, Rockville, MD, United States).

### Statistical Analysis

**FIGURE 1:**
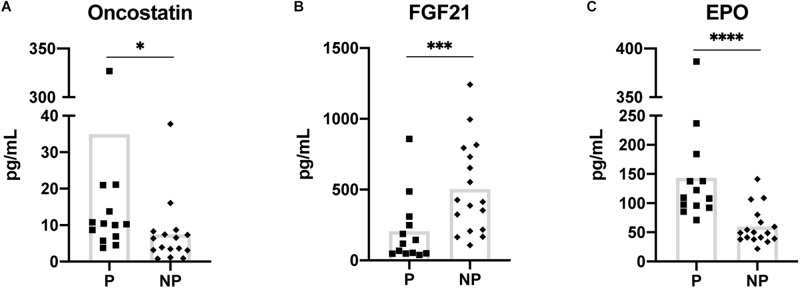
Myokines showing a statistically significant difference in baseline (pre-exercise) serum concentration between pregnant and non-pregnant women **(A–C)**. Means are shown with box on graphs. P, Pregnant; NP, Non-pregnant. ^∗^*p* ≤ 0.05; ^∗∗∗^*p* ≤ 0.001; ^∗∗∗∗^*p* ≤ 0.0001.

**FIGURE 2:**
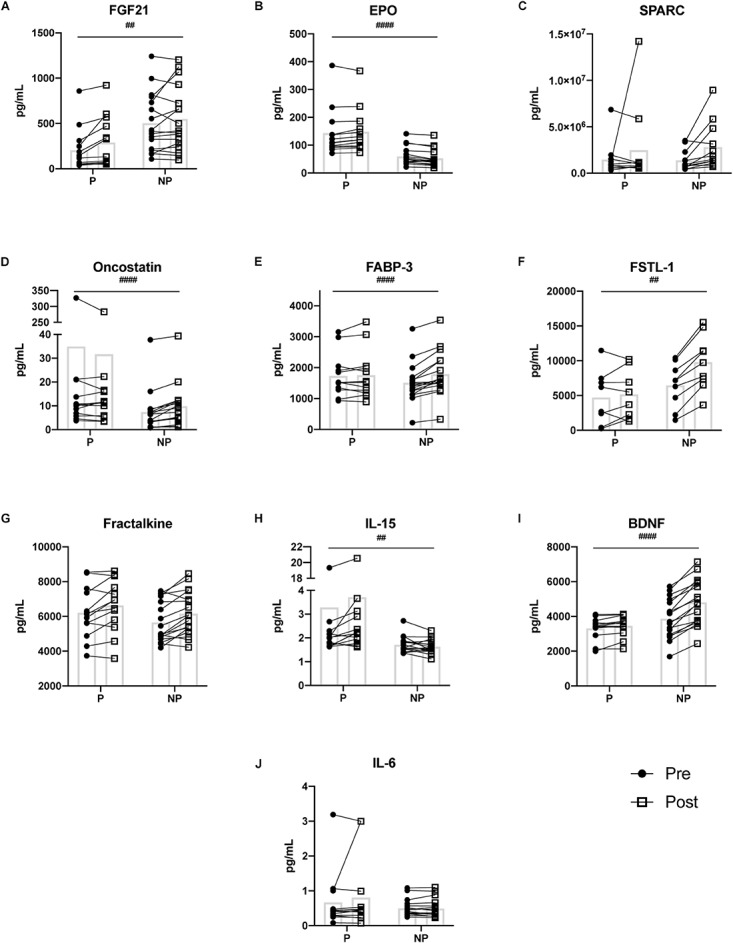
Pre- and post-exercise serum concentrations of ten myokines **(A–J)** measured in serum of pregnant and non-pregnant women. Statistically significant pregnancy status by time interactions are depicted with ‘#’ symbol. Means are shown with box on graphs. P, Pregnant; NP, Non-pregnant; Pre, Pre-exercise serum; Post, Post-exercise serum. ##*p* ≤ 0.01; ###*p* ≤ 0.001; ####*p* ≤ 0.0001.

**FIGURE 3:**
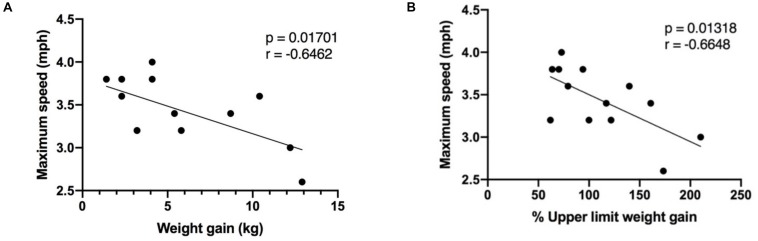
Correlation between maximum speed reached during the exercise session, in pregnant women (*n* = 13), and **(A)** the weight gained at the time of the visit; **(B)** the percentage of upper limit weight gained in accordance to IOM gestational weight gain guidelines. Mph, miles per hour; kg, kilograms.

The Student’s *t*-test and the Mann-Whitney *U* test were used, as appropriate, to compare demographic variables and exercise session indices between pregnant and non-pregnant women. Based on the distribution of the data, either a parametric or non-parametric test was chosen. The main analysis was performed using a 2-way mixed ANOVA to assess changes in myokine concentrations in pregnant compared to non-pregnant women following an acute bout of moderate-intensity walking (Figure 2). Data shown to deviate from normality following a Shapiro-Wilk test for normality were transformed using the natural logarithm. A Bonferroni *post hoc* correction for multiple comparisons was performed. Myokine data were excluded if they were not in the assay’s detectable range. Pearson correlations were performed between delta change in myokine levels and BMI and age, respectively (data not shown), in addition to the exercise session indices and baseline characteristics of participants (Figure 3). For all statistical analyses, *p* ≤ 0.05 was considered significant. Data are presented as mean ± standard deviation (SD). Data presented in Table 1 and Figure 3 were analyzed using GraphPad Prism Software (version 8.0.0, San Diego, CA, United States) while SPSS Software (version 13, Armonk, NY, United States) was used to analyze data in Table 2 and Figures 1-3.

**TABLE 1:** Study population demographics and exercise session indices.

	**Pregnant *N* = 13**	**Non-pregnant *N* = 17**	***p*-value**
Age (years)	31.2 ± 3.5	30.2 ± 4.3	0.48
Gestational age (weeks)	20.1 ± 5.0	–	–
Gestational weight gain at time of session (kg)	5.8 ± 3.9	–	–
Height (cm)	166.7 ± 5.4	166.3 ± 6.3	0.87
Non/pre-pregnant BMI (kg/m^2^)	23.7 ± 3.6	21.8 ± 2.3	0.09
Non/pre-pregnant body weight (kg)	63.7 ± 9.5	60.0 ± 8.4	0.27
Resting heart rate (bpm)	81.4 ± 14.6	74.3 ± 9.3	0.11
Rate of perceived exertion (Borg Scale)	12.7 ± 1.2	12.7 ± 1.3	0.90
Maximal speed reached (mph)	3.4 ± 0.4	3.8 ± 0.3	0.006^∗^
Average speed (mph)	3.2 ± 0.5	3.6 ± 0.3	0.009^∗^
Duration of exercise session (min)	40 ± 2.1	42 ± 1.6	0.006^∗^

*Values are presented as mean ± standard deviation. BMI, body mass index; bpm, beats per minute; mph, miles per hour; min, minutes; ^∗^, indicates significance.*

**TABLE 2:** Myokines for which there was no statistically significant pregnancy by time interaction, but there was a statistically significant main effect of time.

			**Pregnant**		**Non-pregnant**	
				
**Myokine**	***p*-value**	***F*-value**	**Pre-exercise mean ± SD**	**Post-exercise mean ± SD**	**Pre-exercise mean ± SD**	**Post-exercise mean ± SD**
SPARC	0.005	9.6	1465435 ± 1846666	2491219 ± 4172003	1390862 ± 1083435	2809113 ± 2511117
Fractalkine	<0.0001	20.6	6210 ± 1481	6639 ± 1514	5658 ± 1165	6165 ± 1256

*Pre- and post-exercise mean concentrations of myokines presented in pg/mL. SD, standard deviation.*

## Results

### Baseline Characteristics and Exercise Parameters

In total, 13 pregnant and 17 non-pregnant women met the inclusion criteria and were included in the analysis. Baseline characteristics, such as age, height, non/pre-pregnant BMI, weight, and HR, did not differ between groups (Table 1). The RPE scores were compared between the pregnant and non-pregnant groups and did not differ. Thus, the perceived intensity of the exercise session was viewed equally in both groups and corresponded to moderate-intensity (Norton et al., 2010; Berghella and Saccone, 2017). Based on the same relative moderate-intensity exercise session, non-pregnant women were able to reach a significantly higher maximal (*p* = 0.006) and average speed (*p* = 0.009) compared to their pregnant counterparts (Table 1). Additionally, the duration of the exercise session was longer for non-pregnant compared to pregnant women (*p* = 0.006) (Table 1).

### Comparison of Baseline Serum Myokine Levels Between Pregnant and Non-pregnant Women

The 2-way mixed ANOVA revealed differences in baseline serum myokine concentrations between the pregnant and non-pregnant groups. Of the 10 myokines, EPO (*p* ≤ 0.001) and oncostatin (*p* = 0.02) were significantly increased in the pregnant group while FGF21 (*p* = 0.001) was higher in the non-pregnant group (Figure 1).

### Myokine Serum Levels Pre- versus Post-exercise in Pregnant and Non-pregnant Groups

The 2-way mixed ANOVA revealed a significant interaction between time (pre- and post-exercise) and pregnancy status (pregnant and non-pregnant) for FGF21 (*F* = 11.25, *p* = 0.002), EPO (*F* = 18.45, *p* < 0.0001), oncostatin (*F* = 20.56, *p* < 0.0001), FABP-3 (*F* = 20.29, *p* < 0.0001), FSTL-1 (*F* = 20.29, *p* = 0.002), IL-15 (*F* = 10.18, *p* = 0.003), and BDNF (*F* = 35.89, *p* < 0.0001) (Figure 2). Bonferroni corrections were applied to multiple comparisons. Briefly, for the pregnant group, FGF21 (*p* ≤ 0.001), EPO (*p* = 0.004), IL-15 (*p* = 0.018), and BDNF (*p* = 0.025) increased post-exercise (Table 3). Whereas, oncostatin (*p* ≤ 0.001), FABP-3 (*p* ≤ 0.001), FSTL-1 (*p* ≤ 0.001), and BDNF (*p* ≤ 0.001) increased post-exercise in the non-pregnant group, while EPO (*p* = 0.002) decreased post-exercise (Table 3). The main effect of time on SPARC (*F* = 9.60, p = 0.005) and fractalkine (*F* = 20.6, *p* < 0.0001) was significant: women, regardless of pregnancy status, exhibited an increase in both myokines post-exercise (Tables 2, 3).

**TABLE 3:** Post-exercise response of myokines detected in serum of pregnant and non-pregnant women following the acute bout of treadmill walking.

**Myokine**	**Pregnant women**	**Non-pregnant women**	**All women (regardless of pregnancy status)**
**FGF21**	**↑**	**–**	**n/a (i)**
**EPO**	**↑**	**↓**	**n/a (i)**
*SPARC*	*n/a (n/i)*	*n/a (n/i)*	**↑**
**Oncostatin**	**–**	**↑**	**n/a (i)**
**FABP-3**	**–**	**↑**	**n/a (i)**
**FSTL-1**	**–**	**↑**	**n/a (i)**
*Fractalkine*	*n/a (n/i)*	*n/a (n/i)*	**↑**
**IL-15**	**↑**	**–**	**n/a (i)**
**BDNF**	**↑**	**↑**	**n/a (i)**

*Post-exercise response of myokines with a statistically significant pregnancy status and time interaction with subsequent multiple comparison analysis (FGF21, EPO, Oncostatin, FABP-3, FSTL-1, IL-15, and BDNF) are depicted in bold font. Myokines without a significant interaction but with a statistically significant time effect, pre- versus post-exercise (SPARC and fractalkine), are depicted in italic font. **↑**, increase; **↓**, decrease; –, no significant change; n/a (i), not applicable because of statistically significant interaction; n/a (n/i), not applicable because absence of statistically significant interaction.*

### Correlations Between Maximum Speed Reached, and Weight Gained

The following indices were examined to help understand the difference in maximal and average speed reached during the exercise session in the pregnant and non-pregnant groups: age, RHR, pre-pregnancy BMI, gestational age and weight gained at the time of the visit in the pregnant group. There was an inverse correlation between weight gained (expressed both as absolute weight gained, and the percentage of upper limit weight gained according to IOM GWG guidelines) and maximal speed reached (Figure 3). Age, RHR, BMI, and gestational age were not significantly correlated with maximal speed reached or average speed achieved during the exercise session.

## Discussion

Following an acute bout of moderate-intensity walking, the concentration of three myokines; FGF21, EPO, and IL-15 significantly increased in pregnant women. The myokines FABP-3, FSTL-1, and oncostatin exhibited an increase in the non-pregnant group for the same relative intensity exercise. In contrast, EPO was the only myokine to decrease significantly post-exercise in the group of non-pregnant women. SPARC, fractalkine and BDNF were found to increase post-exercise in all women, regardless of pregnancy status. However, BDNF showed a stronger response in non-pregnant women. To our knowledge, this is the first study examining the myokine profile in pregnant women after an acute bout of exercise. Maternal, fetal, and placental health is optimized in women that engage in PA throughout their pregnancies, yet, how PA confers these observed benefits remains to be elucidated. Myokines may be one of the many mediators at play, hence, characterizing the myokine response post-exercise is an essential first step in understanding whether myokines may facilitate the health benefits resulting from PA engagement in pregnancy.

One of the myokines found to differ, FGF21, is a protein thought to regulate energy metabolism by increasing insulin sensitivity and glucose uptake in skeletal muscle and adipocytes (Ge et al., 2011; Mashili et al., 2011; Salminen et al., 2017). Based on our data illustrating an exercise-induced increase in circulating FGF21 levels in pregnant women, we hypothesize that this myokine may enhance glucose uptake and be an important mediator in decreasing the risk of developing gestational diabetes mellitus (GDM; glucose intolerance and insulin resistance in pregnancy). In support of this hypothesis, research shows that exercise interventions decrease the odds of developing GDM (Davenport et al., 2018c).

Increases in circulating EPO, a molecule known to stimulate erythropoiesis (Byts and Sirén, 2009), as a result of exercise in pregnancy, may be playing a role in blood volume adaptation. Blood volume can increase up to 50% during pregnancy compared to the pre-pregnancy period (Soma-Pillay et al., 2016). Thus, elevated EPO may be necessary to induce an increase in red blood cell production. On the contrary, BDNF is a molecule that plays an important role in the regulation of the nervous system as it is in part responsible for the maintenance, development, and survival of neuronal cells (Pedersen, 2009). Low circulating levels of BDNF have been associated with a plethora of diseases such as obesity, type 2 diabetes, depression, and cognitive impairments (Pedersen, 2009). In the context of pregnancy, low serum BDNF has been associated with antenatal depression (Fung et al., 2015) and increased risk of low birth weight (Christian et al., 2016). Increased levels of BNDF, made possible by exercise, may be important to promote the health of the nervous system.

Of the other myokines shown to increase in pregnant women post-exercise, IL-15 is known to increase trophoblast invasion and migration (Zygmunt et al., 1998). Moreover, IL-15 has been shown to play a role in muscle growth (Brandt and Pedersen, 2010) and the reduction of adipose tissue mass (Nielsen and Pedersen, 2008). Thus, increased IL-15 following exercise may help facilitate appropriate GWG corresponding to the IOM guidelines (American College of Obstetricians and Gynecologists, 2013). In summary, the increase in certain myokines following exercise may contribute to the optimization of maternal-fetal health and future work should aim to explore this hypothesis.

Brisk walking was chosen as the exercise modality for this study as it is a recommended moderate-intensity PA during pregnancy (Mottola et al., 2018). Since the walking session was of ‘relative’ intensity and our groups did not differ based on anthropometric and baseline characteristics (Table 1), it is particularly interesting that both groups demonstrated different myokine response profiles. A possible explanation as to why pregnant women are not exhibiting a change in certain circulating myokines compared to the non-pregnant group following a bout of MPA is the speed at which they were walking. Non-pregnant women were able to reach a higher maximal speed and averaged a higher speed than their non-pregnant counterparts, for the same relative intensity (Table 1). Furthermore, as illustrated in Figure 3, maximal speed reached in the pregnant group was inversely correlated to both GWG and percentage of upper-limit of recommended GWG, based on the IOM guidelines (Committee on Obstetric Practice, 2014). These results suggest that although the exercise session was of comparable relative intensity, and that the pregnant and non-pregnant groups did not differ in variables that would influence target HR zone intensity, such as age and RHR, the weight that pregnant women gain across pregnancy is likely hindering their ability to reach the higher speeds. Thus, it is possible that lower speed would translate to an insufficient muscle fiber recruitment or stimulation needed to elicit an observable change in a more substantial number of circulating myokines, in the pregnant participants. It is also conceivable that a higher intensity is necessary to stimulate myokine synthesis and release. For instance, circulating IL-6 is consistently shown to increase post-exercise, however, in this study, it remains unchanged in both the pregnant and non-pregnant groups following the walking session which may be attributed to the intensity of the exercise (Leal et al., 2018; Garneau and Aguer, 2019).

The myokine secretome is vast, complex, and the release of distinct myokines seem to be dependent on specific muscular stimulus. In light of this complexity, studies examining the same myokine report contradictory results. For instance, serum secreted protein acidic and rich in cysteine (SPARC) levels have been showed to increase following a 30-min aerobic bout of cycling in healthy young men (Aoi et al., 2013). In contrast, reports indicate no change in serum SPARC levels following a brief bout of supramaximal cycle sprint (Songsorn et al., 2017). These discrepancies are likely related to variations in experimental protocols. Exercise type or modality, intensity, and duration are all factors that could influence the rate of synthesis and thus, the release of detectable myokines in the bloodstream (Leal et al., 2018). Variables such as nutritional status, environmental conditions (Rai and Demontis, 2015) and disease states (Kurdiova et al., 2014) may also be other elements influencing myokine response. The duration of the exercise session in this study is one variable that may be contributing to the differing myokine response post-exercise between both groups. Pregnant women exercised for a significantly shorter period (40-min vs. 42-min) compared to non-pregnant women (Table 1). Although this result was not by design, it indicates that the acclimation phase, during which the speed is increased every 1-min interval until the target heart rate range is met, was shorter for pregnant women who reached the desired intensity more rapidly. Thus, some myokines may require a longer duration rather than a higher intensity of exercise in order to be synthesized and released into the bloodstream.

While exercise session parameters may account for a proportion of the difference observed in the myokines released by the pregnant compared to the non-pregnant women, pregnancy-specific responses could also be at play. For instance, FGF21 increases exclusively in the pregnant group following the acute bout of exercise. Among the many physiological adaptions during pregnancy, cardiac hypertrophy occurs thereby accommodating the transient increase in blood volume (Li et al., 2012; Redondo-Angulo et al., 2017). Redondo-Angulo and colleagues explain that FGF21 is a key molecule responsible for cardiac remodeling during pregnancy. Thus, the increase of some and lack of change in other myokines in the pregnant compared to the non-pregnant women may have implications related to the physiological adaptations required as a result of pregnancy.

We also compared baseline myokine levels in the pregnant and the non-pregnant participants, as differences in baseline concentrations could potentially help clarify the main result. Of the 10 myokines studied, three demonstrated differences between pregnant and non-pregnant at baseline. Interestingly, pregnant participants had higher baseline levels of EPO and oncostatin, while FGF21 was lower. Regarding oncostatin, our results are consistent with research by Ogata et al. (2000) who demonstrated a higher level of serum oncostatin in pregnant versus non-pregnant women, attributed to production by decidual and chorionic tissues (Ogata et al., 2000). In line with these findings, oncostatin has been identified as a member of a cytokine family that plays a role in cellular differentiation (Rose and Bruce, 1991), a vital cellular process during pregnancy (Malassiné and Cronier, 2002) which could clarify the increased levels observed in pregnant individuals. Also, the known elevation in blood volume during pregnancy provides logical reasoning for higher levels of EPO in pregnant women. In brief, although myokines are synthesized and secreted by skeletal muscle; similar molecules are also regulated via other tissues and organs, regardless of PA engagement. Likewise, cardiovascular adaptions during pregnancy are particularly relevant when considering serum myokine levels. Blood volume increases disproportionately during pregnancy as plasma volume exhibits a more significant increase compared to red blood cell mass, creating a concept known as hemodilution (Costantine, 2014; Soma-Pillay et al., 2016). Thus, it is possible that blood volume adaptations and fluctuations in the serum proteome across gestation may influence observed myokine levels in pregnant women (Romero et al., 2017).

Pregnancy is undoubtedly a critical period for the health of both the fetus and the mother. While it is well established that maternal PA is essential for the short- and long- term health of mom and baby, the pathways and mediators involved in the crosstalk between skeletal muscle, the placenta and subsequently the fetus are mostly unknown. The results of this study propose four possible mediators: FGF21, EPO, BDNF, and IL-15. Recognizing the preliminary nature of this work focusing on a panel of well-characterized myokines, other myokines are likely being released during pregnancy that were not examined, and various exercise parameters may elicit differing circulatory responses in this particular population.

### Limitations

Although our study had a similar sample size to other studies examining myokines post-exercise, such as six (Steensberg et al., 2002) and nine males (Aoi et al., 2013), given the inter-individual variability in our pregnant group, a larger sample size could potentially translate to differences in myokines that remained unchanged. However, the lack of information and published results on myokines in pregnant women did not allow us to perform a power calculation to determine an appropriate sample size. Additionally, a measure of fitness would have been helpful in providing insight as to the role of chronic exercise on myokine release. However, obtaining such a measure in our pregnant population would have required multiple visits and in turn render the study less feasible.

### Future Directions

Our results warrant further investigation of the role of myokines in pregnancy. As this was an initial characterization of the myokine profile following a single bout of moderate-intensity walking, follow-up work is needed to understand whether myokine signaling is a vital part of placental and fetal health optimization. Future studies should aim to investigate whether different exercise modalities, such as cycling, swimming, or resistance training, which are all deemed safe during pregnancy, influence the myokine response in pregnant women. The exercise duration chosen for this study was based on current guidelines for PA during pregnancy (Mottola et al., 2018) and ranged from 36 to 43 min for the pregnant participants. Longer exercise durations or bouts of higher intensity may elicit different responses. Examination of the relationship between objectively measured PA level (volume) during pregnancy and the myokine response would also be valuable. Further exploration of these variables would allow us to identify if prior fitness level or chronic exposure to exercise influences circulating myokines following PA.

## Conclusion

This novel study found that walking at moderate-intensity between 36 and 43 min elicited a change in four of the ten myokines measured in the pregnant participants and five of the ten myokines in non-pregnant controls, while two myokines increased post-exercise regardless of pregnancy status. Future studies should aim to explore whether the myokines shown to be elevated post-exercise in the pregnant group of this study are involved in the molecular mechanism by which maternal, fetal and placental health is optimized as a result of PA engagement.

## Data Availability Statement

All datasets generated for this study are included in the manuscript/supplementary files.

## Ethics Statement

This study was carried out in accordance with the recommendations of the Research Ethics Board at the University of Ottawa (file number: H-06-18-634) with written informed consent from all subjects. All subjects gave written informed consent in accordance with the Declaration of Helsinki. The protocol was approved by Research Ethics Board at the University of Ottawa.

## Author Contributions

KH drafted the manuscript. KH and SM primarily performed data collection and designed the study. KM, LG, and CA secondarily performed the data collection. All authors contributed to the design of the study, revised and edited the manuscript, and read and approved the final version of the manuscript.

## Conflict of Interest

The authors declare that the research was conducted in the absence of any commercial or financial relationships that could be construed as a potential conflict of interest.

## Abbreviations

BDNF: brain-derived neurotophic factor
BMI: body mass index
EPO: erythropoietin
EV: extracellular vesicles
FABP-3: fatty acid binding protein 3
FGF21: fibroblast growth factor 21
FSTL-1: follistatin-like 1
GDM: gestational diabetes mellitus
GLT: godin leisure time
GWG: gestational weight gain
HR_max_: heart rate maximum
HR: heart rate
HRR: heart rate reserve
IOM: Institute of Medicine
LIF: leukemia inhibitory factor
MPA: moderate-intensity physical activity
PA: physical activity
RHR: resting heart rate
SPARC: secreted protein acidic and rich in cysteine.
